# The R3-Type MYB Transcription Factor BrMYBL2.1 Negatively Regulates Anthocyanin Biosynthesis in Chinese Cabbage (*Brassica rapa* L.) by Repressing MYB–bHLH–WD40 Complex Activity

**DOI:** 10.3390/ijms23063382

**Published:** 2022-03-21

**Authors:** JiYeon Kim, Da-Hye Kim, Jong-Yeol Lee, Sun-Hyung Lim

**Affiliations:** 1 Division of Horticultural Biotechnology, School of Biotechnology, Hankyong National University, Anseong 17579, Korea; kjy110395@gmail.com (J.K.); kimdh143@naver.com (D.-H.K.); 2 Research Institute of International Technology and Information, Hankyong National University, Anseong 17579, Korea; 3 National Institute of Agricultural Sciences, Rural Development Administration, Jeonju 54874, Korea

**Keywords:** anthocyanin, BrMYBL2.1, Chinese cabbage, insertional mutation, repressor, ubiquitination

## Abstract

Chinese cabbage (*Brassica rapa* L.) leaves are purple in color due to anthocyanin accumulation and have nutritional and aesthetic value, as well as antioxidant properties. Here, we identified the R3 MYB transcription factor BrMYBL2.1 as a key negative regulator of anthocyanin biosynthesis. A Chinese cabbage cultivar with green leaves harbored a functional BrMYBL2.1 protein, designated BrMYBL2.1-G, with transcriptional repressor activity of anthocyanin biosynthetic genes. By contrast, BrMYBL2.1 from a Chinese cabbage cultivar with purple leaves carried a poly(A) insertion in the third exon of the gene, resulting in the insertion of multiple lysine residues in the predicted protein, designated BrMYBL2.1-P. Although both BrMYBL2.1 variants localized to the nucleus, only BrMYBL2.1-G interacted with its cognate partner BrTT8. Transient infiltration assays in tobacco leaves revealed that BrMYBL2.1-G, but not BrMYBL2.1-P, actively represses pigment accumulation by inhibiting the transcription of anthocyanin biosynthetic genes. Transient promoter activation assay in Arabidopsis protoplasts verified that BrMYBL2.1-G, but not BrMYBL2.1-P, can repress transcriptional activation of *BrCHS* and *BrDFR*, which was activated by co-expression with BrPAP1 and BrTT8. We determined that BrMYBL2.1-P may be more prone to degradation than BrMYBL2.1-G via ubiquitination. Taken together, these results demonstrate that BrMYBL2.1-G blocks the activity of the MBW complex and thus represses anthocyanin biosynthesis, whereas the variant BrMYBL2.1-P from purple Chinese cabbage cannot, thus leading to higher anthocyanin accumulation.

## 1. Introduction

Chinese cabbage (*Brassica rapa* L.), a member of the *Brassicaceae* family, is an economically important vegetable crop grown in Asian countries. Different Chinese cabbage cultivars have green or purple leaves, depending on the accumulation and distribution of anthocyanin and chlorophyll pigments. Anthocyanins are flavonoid-derived metabolites that confer red, purple, or blue coloration to leaves, fruits, and flowers of many plant species [1]. Anthocyanins act as visual attractants for pollinators or seed dispersers and help to protect plants against various biotic and abiotic stresses [2,3]. In addition, anthocyanins have antioxidant properties and have thus attracted widespread interest due to their health benefits in preventing chronic human diseases, certain cancers, and cardiovascular diseases [4,5,6].

The anthocyanin biosynthetic pathway is well understood and most genes encoding the relevant enzymes and transcriptional regulators have been identified in many plant species. These biosynthetic genes are regulated by R2R3 MYB transcription factors (TFs), basic helix-loop-helix (bHLH) TFs, and WD40 repeat proteins, which interact to form MYB–bHLH–WD40 (MBW) complexes [7,8]. The MYB TFs of the MBW complexes have the two conserved imperfect repeats (R2 and R3) at their N-termini for binding DNA and a variable region at their C-termini for regulating transcription [9,10]. Flavonoid-related bHLH TFs form transcriptional complexes at the promoters of anthocyanin biosynthetic genes by interacting with their R2R3 MYB partners [11,12,13,14]. Finally, the WD40 protein acts as a scaffold onto which the MBW complex is assembled and stabilizes the interaction between the MYB and bHLH TFs, rather than having a direct regulatory function [7,15].

In addition to MYB activators, several active or passive MYB repressors participate in regulating flavonoid biosynthesis. Active MYB repressors contain a highly conserved bHLH-interacting motif (BIM) at their N-termini and an ethylene-responsive element binding factor-associated amphiphilic repression (EAR) motif (pdLNL(D/E)L or DLNxxP) or a TLLLFR motif at their C termini. These active repressors directly downregulate expression of the genes targeted by the anthocyanin-activating MBW complex [16,17]. Passive MYB repressors, by contrast, do not have EAR or TLLLFR motifs, but do contain a BIM; this suggests that passive MYB repressors act by sequestering bHLH proteins from anthocyanin-activating MBW complexes [16,18].

Chinese cabbage leaves do not typically accumulate anthocyanins, due to incomplete activation of the flavonoid biosynthetic pathway. However, anthocyanin biosynthesis can be induced by several TFs, including the R2R3 MYB BrMYB2, PRODUCTION OF ANTHOCYANIN PIGMENT1 (BrPAP1), and the bHLH TRANSPARENT TESTA8 (BrTT8) [19,20,21,22,23,24]. Despite much recent progress, the molecular mechanism underlying the coloration of Chinese cabbage remains unknown. Therefore, to investigate the anthocyanin biosynthetic pathway in more detail, we analyzed the expression level of flavonoid biosynthetic genes and regulators, and anthocyanin accumulation, in differently colored Chinese cabbage seedlings grown under various cultivation conditions. By examining the transcriptomes of these seedlings, we were able to isolate and sequence the regulatory R3 MYB repressor MYB-LIKE2.1 (BrMYBL2.1). This revealed that the *BrMYBL2.1* (*BrMYBL2.1-P*) from the purple cultivar contains a poly(A) insertion causing multiple lysine residues in the predicted protein. Using yeast two-hybrid assays, we also demonstrate that BrMYBL2.1-G can interact with the BrTT8 of the anthocyanin-activating MBW complex, but BrMYBL2.1-P cannot. Furthermore, through transient infiltration assays in tobacco (*Nicotiana tabacum*) leaves, we showed that BrMYBL2.1-G negatively regulates anthocyanin biosynthesis by inhibiting MBW complex activity, thus limiting pigment production; disruption of this negative regulation in purple Chinese cabbage allows pigment production.

## 2. Results

### 2.1. BrMYBL2.1 Is Highly Expressed in Green Chinese Cabbage

**Figure 1:**
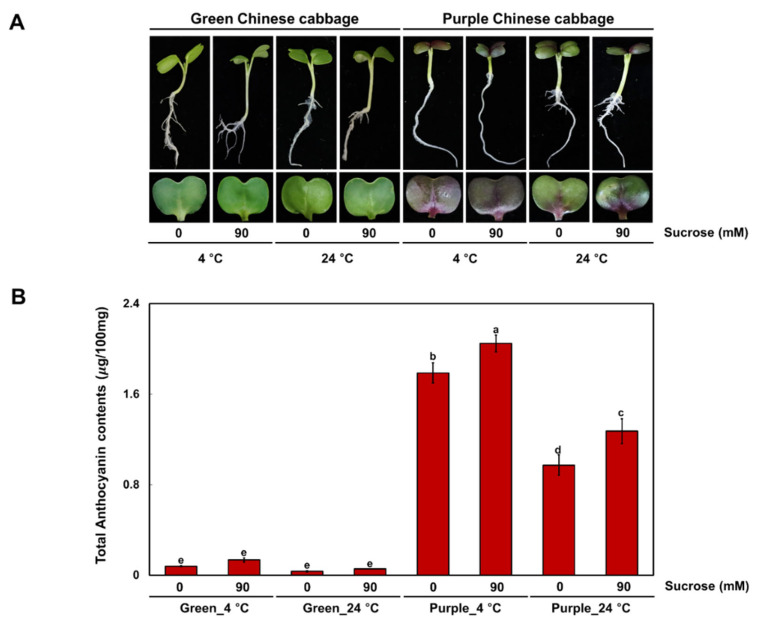
Phenotype and anthocyanin contents of green and purple Chinese cabbage under various cultivation conditions. (**A**) Phenotypes of seedlings from two Chinese cabbage cultivars were grown under low temperature or an optimal temperature, with or without 90 mM sucrose. (**B**) Mean anthocyanin contents of seedlings from green and purple Chinese cabbage cultivars. Results represent the mean values ± standard deviation (SD). Three independent biological replicates were performed. Different letters indicate significantly different values (*p* < 0.01), as determined by two-way ANOVA followed by Duncan’s multiple range tests.

We grew Chinese cabbage seedlings with green (G) or purple (P) leaves at low temperature (4 °C) or at an optimal temperature (24 °C), with or without sucrose. The cotyledons of P seedlings showed purple pigmentation under all treatment conditions, while G seedlings remained green (Figure 1A). In agreement with their visible phenotypes, cotyledons of P seedlings accumulated high levels of anthocyanins under all conditions, whereas the G seedlings accumulated very low levels of anthocyanins (Figure 1B). Exposure to cold temperature increased the anthocyanin contents in all seedlings regardless of the presence of sucrose in the growth medium. Similarly, addition of sucrose to the medium promoted the accumulation of anthocyanins in cotyledons of seedlings grown at 4 °C and 24 °C. We concluded that anthocyanin accumulation determines the pigmentation phenotype of these Chinese cabbage seedlings.

**Figure 2:**
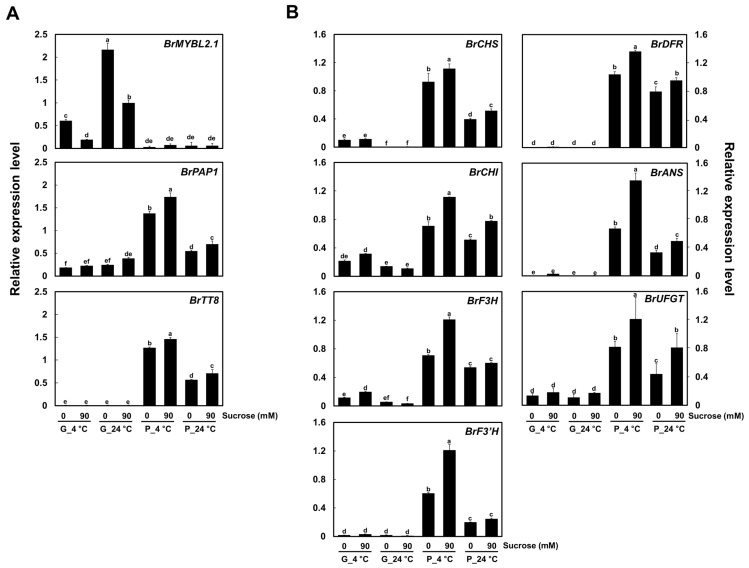
Expression of *BrMYL2.1* and other anthocyanin regulatory and biosynthetic genes under various cultivation conditions in green and purple Chinese cabbage. (**A**) Relative transcript levels of the anthocyanin regulatory genes. (**B**) Relative transcript levels of the anthocyanin biosynthetic genes. G, green Chinese cabbage; P, purple Chinese cabbage. Results represent the means ± SD from three independent biological replicates. *BrEF1α* was used as the reference gene. Different letters indicate significantly different values (*p* < 0.01), as determined by two-way ANOVA followed by Duncan’s multiple range tests.

We examined the expression levels of the genes encoding the three regulators related to anthocyanin biosynthesis, *BrMYBL2.1*, *BrPAP1*, and *BrTT8*, by reverse transcription-quantitative PCR (RT-qPCR) in seedlings of the two Chinese cabbage cultivars grown at 4 °C or 24 °C, in the presence or absence of sucrose in the growth medium. *BrPAP1* and *BrTT8* were highly expressed in P seedlings and their expression levels reflected the extent of pigment accumulation (Figure 2A). *BrMYBL2.1*, an ortholog of Arabidopsis *AtMYBL2*, was highly expressed in G seedlings in all growth conditions, but was poorly expressed in P seedlings. The expression of *BrMYBL2.1* thus followed an opposite pattern to that of anthocyanin contents. 

Flavonoid biosynthetic genes were usually expressed to much higher levels in P seedlings than in G seedlings (Figure 2B). Expression of the early biosynthetic genes *CHALCONE SYNTHASE* (*BrCHS*), *CHALCONE ISOMERASE* (*BrCHI*), *FLAVANONE 3-HYDROXYLASE* (*BrF3H*), and *FLAVONOID 3′-HYDROXYLASE* (*BrF3′H*) was high in P seedlings, but usually low to very low in G seedlings. Likewise, transcript levels of the late biosynthetic genes *DIHYDROFLAVONOL 4*-*REDUCTASE* (*BrDFR*), *ANTHOCYANIDIN SYNTHASE* (*BrANS*), and *UDP-GLUCOSE: FLAVONOID 3-O-GLUCOSYLTRANSFERASE* (*BrUFGT*) were high in P seedlings, in agreement with the accumulation of anthocyanins. Taken together, these results confirmed that anthocyanin contents are well coincided with the expression levels of flavonoid biosynthetic genes and the active regulators, *BrPAP1* and *BrTT8*, and reflect an opposite pattern of *BrMYBL2.1* expression, across various cultivation conditions.

### 2.2. Phylogenetic Analysis of BrMYBL2.1 and MYB Repressors 

To explore the mechanism underlying the difference in anthocyanin biosynthesis between G and P Chinese cabbage cultivars, we amplified cDNA sequences for *BrMYBL2.1*, *BrPAP1*, and *BrTT8* from each cultivar. Primers were designed based on previously reported sequences [20,23,24]. The cDNA sequence of *BrMYBL2.1* from the G cultivar was 100% identical to the previously reported sequence of *BrMYBL2.1* (GenBank accession number JN379103): it comprised a 591-bp coding sequence encoding a predicted protein of 196 amino acids, which we refer to hereafter as a *BrMYBL2.1-G* (GenBank accession number OK905440). By contrast, the cDNA sequence of *BrMYBL2.1* from the P cultivar contained a poly(A) insertion with target site duplication (TSD; GGGAATCGATCCAACT) in the third exon (Supplementary Figure S1, see Supplementary Materials). The 60-nucleotide insertion did not disrupt in reading frame of *BrMYBL2.1*, but resulted in a 651-bp coding sequence encoding a predicted protein of 216 amino acids, with 20 new amino acids, including 13 lysine residues, inserted between the R3 domain and the C2 motif. We therefore designated this variant *BrMYBL2.1-P* (GenBank accession number OK905441). 

The cDNA sequences of *BrPAP1* from the G and P cultivars are 100% identical to the previously reported sequence for *BrPAP1* (GenBank accession number KAG5409581) and *BrPAP1* (GenBank accession number CAG7892910), respectively. Amino acid sequence comparison showed that these two proteins shared 99.6% identity and showed the one amino acid substitution at position 158 (Supplementary Figure S2). The cDNA sequences of *BrTT8* from the G and P cultivars are 100% identical to the previously reported sequence for *BrTT8* (GenBank accession number XM_009115326). 

**Figure 3:**
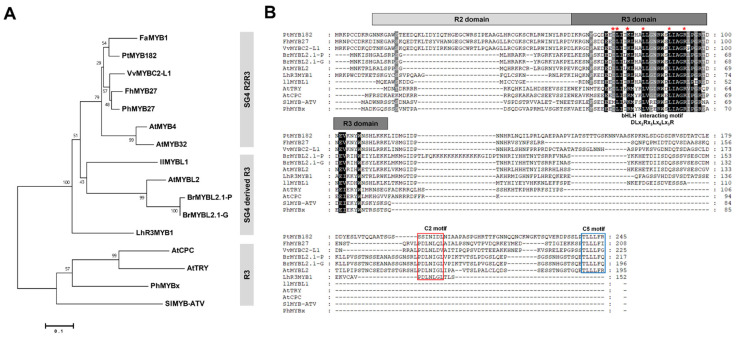
Phylogenetic relationships and multiple sequence alignment among anthocyanin MYB repressors from Chinese cabbage and other plant species. (**A**) Phylogenetic tree of BrMYBL2.1 and other MYB repressors proteins from other plants. The phylogenetic tree was constructed using the neighbor-joining method with MEGA6 software. Numbers next to the nodes are bootstrap values from 1000 replications. The scale bar corresponds to 0.1 amino acid substitutions per site. The following GenBank or Arabidopsis TAIR accession numbers were used: *Arabidopsis thaliana* AtMYB4 (At4g38620), AtMYB32 (At4g34990), AtMYBL2 (At1g71030), AtCPC (At2g46410), AtTRY (At5G53200); *Brassica rapa* BrMYBL2.1-G (OK905440), BrMYBL2.1-P (OK905441); *Fragaria × ananassa* FaMYB1 (AF401220); *Freesia hybrid* FhMYB27 (QJW70308); *Iochroma loxense* IlMYBL1 (ASR83103); *Lilium hybrid* LhR3MYB1 (LC429593); *Petunia × hybrida* PhMYB27 (AHX24372), PhMYBx (AHX24371); *Populus tremuloides* PtMYB182 (KP723392); *Solanum lycopersicum* SlMYB-ATV (MF197509); *Vitis vinifera* VvMYBC2-L1 (JX050227). (**B**) Multiple sequence alignment of BrMYBL2.1s with representative MYB repressors. Absolutely conserved residues are highlighted in black and partial conservation is shown in gray. The R2 and R3 domains are indicated with light gray and dark grey boxes, respectively. The specific motif responsible for interacting with bHLH factors is indicated by the red asterisks. The conserved repression motifs in the C2 and C5 motifs are indicated by the red and blue boxes, respectively.

To determine the relationship between BrMYBL2.1 (BrMYBL2.1-G and BrMYBL2.1-P) and other known MYB repressors of phenylpropanoid and flavonoid biosynthesis, we constructed a phylogenetic tree (Figure 3A). Both BrMYBL2.1 proteins clustered with SG4 derived R3 MYB TFs, including AtMYBL2 from Arabidopsis, IlMYBL1 from Iochroma (*Iochroma loxense*), and LhR3MYB1 from Asiatic lily (*Lilium hybrid*), all of which are negative regulators of anthocyanin biosynthesis [25,26,27,28].

Sequence alignment showed that the predicted BrMYBL2.1-G and BrMYBL2.1-P protein sequences were similar to those of the R3 MYB repressors AtMYBL2 from Arabidopsis and the R2R3 MYB repressors FhMYB27 from freesia (*Freesia hybrida*) [25,26,29]. It indicated that BrMYBL2.1-G and BrMYBL2.1-P harbor the conserved BIM in the R3 domain, which mediates the interaction with bHLH partners (Figure 3B). BrMYBL2.1-G and BrMYBL2.1-P contained additional conserved motifs in their C-terminal regions: the C2 (EAR) motif and C5 (TLLLFR) motif, which have a repressive function [29,30]. These results therefore suggest that both BrMYBL2.1 variants are likely to repress anthocyanin biosynthesis.

### 2.3. BrMYBL2.1-G and BrMYBL2.1-P Localize to the Nucleus

**Figure 4:**
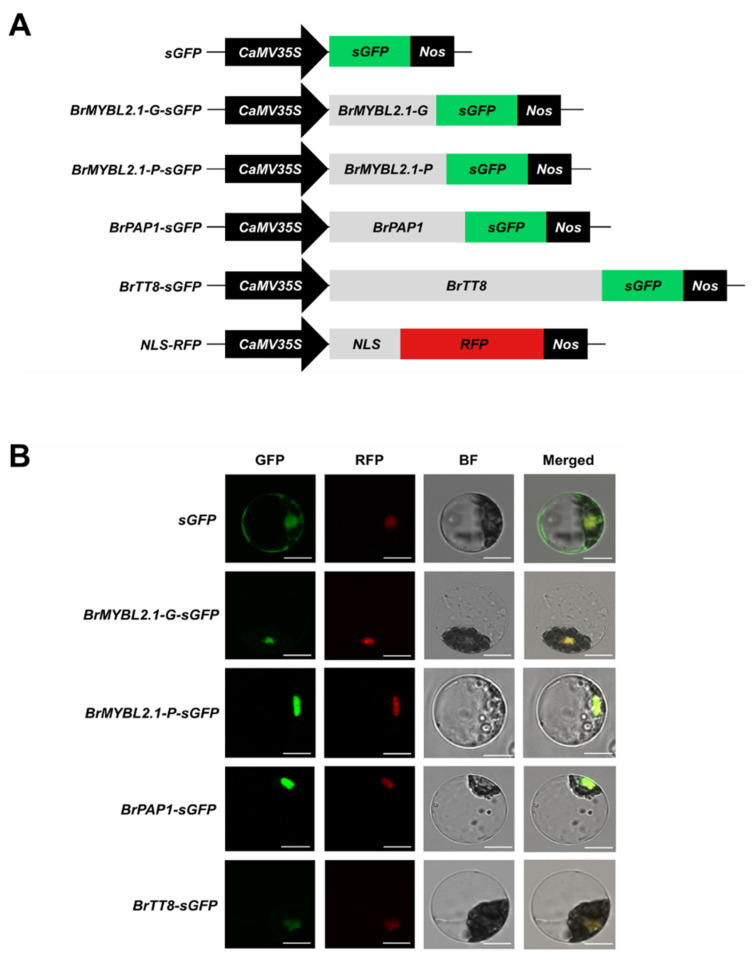
Subcellular localization of BrMYBL2.1-G, BrMYBL2.1-P, BrPAP1, and BrTT8 in Arabidopsis leaf protoplasts. (**A**) Schematic diagram of the constructs used in this experiment: *CaMV35S*, cauliflower mosaic virus *35S* promoter; *sGFP*, soluble green fluorescent protein (*GFP*) gene; *BrMYBL2.1-G-sGFP*, *BrMYBL2.1-G* fused to *sGFP*; *BrMYBL2.1-P-sGFP*, *BrMYBL2.1-P* fused to *sGFP*; *BrPAP1-sGFP*, *BrPAP1* fused to *GFP*; *BrTT8-sGFP*, *BrTT8* fused to *sGFP*; *NLS-RFP*, nuclear localization signal fused to the red fluorescent protein (*RFP*) gene; *Nos*, nopaline synthase terminator. (**B**) In vivo localization pattern of BrMYBL2.1-G, BrMYBL2.1-P, BrPAP1, and BrTT8 in Arabidopsis protoplasts. Representative protoplasts accumulating each fusion protein are shown 16 h after transformation. GFP, GFP fluorescence; RFP, RFP fluorescence; BF, bright field and merged, merged of GFP, RFP, and bright field images. Bars, 10 μm.

Next, we assessed the subcellular distribution of BrMYBL2.1-G, BrMYBL2.1-P, BrPAP1 and BrTT8. Arabidopsis mesophyll protoplasts were transiently transfected with constructs encoding green fluorescent protein (GFP)-fused BrMYBL2.1-G, BrMYBL2.1-P, BrPAP1, or BrTT8, and a construct encoding red fluorescent protein (RFP) fused with the nuclear localization signal (NLS) of the SV40 large T-antigen as a marker of the nucleus (Figure 4A). After transfection, we examined GFP and RFP fluorescence by confocal microscopy. Despite their sequence differences, both BrMYBL2.1 variants localized to the nuclei of transfected protoplasts, as did BrPAP1-GFP and BrTT8-GFP fusions, as evidenced by their co-localization with RFP fluorescence (Figure 4B). We therefore concluded that the anthocyanin regulators BrMYBL2.1, BrPAP1, and BrTT8 are nucleus-localized proteins. This result also suggested that anthocyanin accumulation in the purple Chinese cabbage cultivar cannot be explained by differential localization of BrMYBL2.1-P. Moreover, despite the 20-amino-acid insertion, BrMYBL2.1-P localizes to the nucleus and, therefore, it has the potential to regulate gene expression.

### 2.4. BrMYBL2.1-G, but Not BrMYBL2.1-P, Physically Interacts with BrTT8 

**Figure 5:**
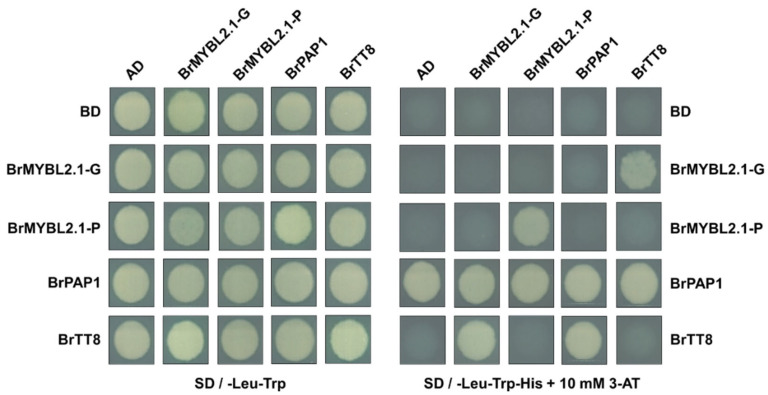
Interaction tests between BrMYBL2.1-G, BrMYBL2.1-P, BrPAP1, and BrTT8 by yeast two-hybrid assay. AD, activation domain; BD, binding domain; SD, synthetic defined medium; 3-AT, 3-amino-1,2,4-triazole; SD − TL, SD medium lacking Trp and Leu; SD − TLH + 3AT, SD medium lacking Trp, Leu, His and containing 10 mM 3-AT.

To understand the role of BrMYBL2.1-G and BrMYBL2.1-P in anthocyanin biosynthesis, we used a yeast two-hybrid assay. We individually cloned the coding sequences of *BrMYBL2.1-G*, *BrMYBL2.1-P*, *BrPAP1*, and *BrTT8* into the vectors pGBKT7 and pGADT7, which harbor the GAL4 DNA-binding domain (BD) or the GAL4 DNA-activation domain (AD), respectively. We introduced appropriate pairs of constructs into strain MaV203 and grew the transformants on a selective synthetic-defined (SD) medium containing 10 mM 3-amino-1,2,4-triazole, a competitive inhibitor of HIS3. These assays showed that BrTT8 physically interacts with BrPAP1 and with BrMYBL2.1-G, but not with BrMYBL2.1-P (Figure 5). Full-length BrPAP1 exhibited autoactivation activity when fused to BD, as often observed with R2R3 MYB TFs [31]. Interestingly, BrMYBL2.1-P did not have this autoactivation function, but did demonstrate self-interaction in vivo. This indicates that BrMYBL2.1-P probably functions as a homodimer in the regulation of anthocyanin biosynthesis. Taken together, these results suggest that BrMYBL2.1-G might disrupt the formation of the anthocyanin-activating MBW complex by physically interacting with BrTT8, and act as a negative regulator of anthocyanin biosynthesis. By contrast, BrMYBL2.1-P lacks the ability to interact with BrTT8 and therefore may lose the ability to act as a negative regulator on anthocyanin biosynthesis. 

### 2.5. BrMYBL2.1-G Represses Anthocyanin Biosynthesis in Tobacco Leaves

**Figure 6:**
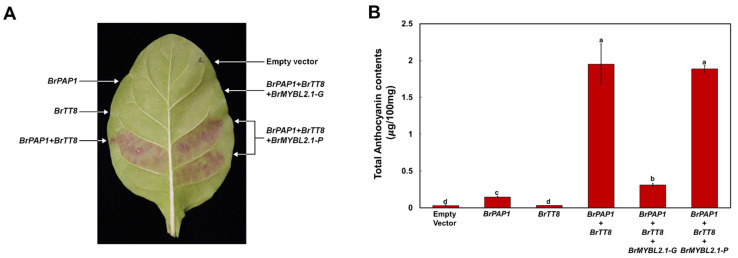
Functional analysis of *BrMYBL2.1-G* and *BrMYBL2.1-P* for anthocyanin biosynthesis, as a repressor. Visual phenotype and anthocyanin contents in tobacco leaves transiently infiltrated with Agrobacterium strains harboring an empty vector or constructs *BrPAP1*, *BrTT8*, *BrMYBL2.1-G*, and *BrMYBL2.1-P* in the indicated combinations. (**A**) Representative photograph of a transiently infiltrated tobacco leaf 5 days after agroinfiltration. (**B**) Anthocyanin contents of the leaf sections shown in (**A**). Results are the means ± SD from three independent biological replicates. Different letters indicate significantly different values (*p* < 0.01), as determined by one-way ANOVA followed by Duncan’s multiple range tests.

Previous studies revealed that simultaneous accumulation of R2R3 MYB and bHLH TFs enhances anthocyanin accumulation in various plants, including Arabidopsis, apple (*Malus domestica*), chrysanthemum (*Chrysanthemum × morifolium*), radish (*Raphanus sativus*), rice (*Oryza sativa*), and tobacco (*Nicotiana tabacum*) [12,13,32,33,34,35]. In Chinese cabbage, the R2R3 MYB TFs BrPAP1 and BrMYB2 and the bHLH TF BrTT8 are reportedly involved in anthocyanin biosynthesis, although direct evidence is lacking [21,36]. To explore the role of *BrPAP1* and *BrTT8* in anthocyanin biosynthesis, we conducted a transient expression assay in tobacco leaves. Infiltration of tobacco leaves with a construct expressing BrPAP1 alone could slightly trigger anthocyanin accumulation, but expressing BrTT8 alone did not trigger anthocyanin accumulation (Figure 6). However, co-infiltration of *BrPAP1* and *BrTT8* resulted in purple pigmentation of the infiltrated sectors. We measured the anthocyanin contents in leaf discs collected at 5 days post infiltration (Figure 6B). These results indicated that simultaneous expression of *BrPAP1* and *BrTT8* activates anthocyanin biosynthesis. 

To assess the repressor function of BrMYBL2.1 variants in anthocyanin biosynthesis, we co-infiltrated tobacco leaves with BrPAP1, BrTT8, and BrMYBL2.1-G or BrMYBL2.1-P. The expression of BrMYBL2.1-G inhibited the accumulation of pigments when co-infiltrated with BrPAP1 and BrTT8, as reflected visually in leaf sectors (Figure 6A) and after extraction of anthocyanins from infiltrated areas (Figure 6B). By contrast, the co-infiltration of BrPAP1 and BrTT8 with BrMYBL2.1-P failed to inhibit pigment accumulation (Figure 6A,B).

**Figure 7:**
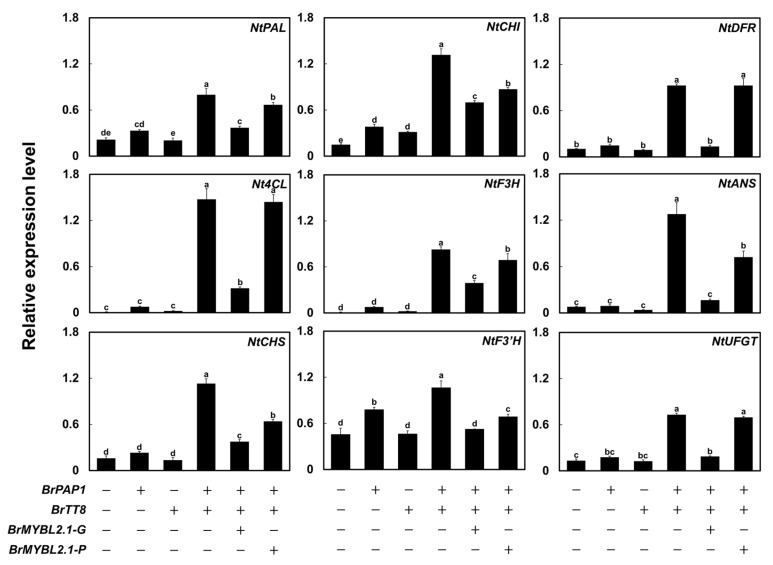
Relative transcript levels of anthocyanin biosynthetic genes in tobacco leaves transiently infiltrated with *BrMYBL2.1-G* or *BrMYBL2.1-P* constructs. Relative transcript levels were determined by RT-qPCR, with *NtGAPDH* used as a reference gene. Results are the means ± SD from three independent biological replicates. Different letters indicate significantly different values (*p* < 0.01), as determined by one-way ANOVA followed by Duncan’s multiple range tests.

We measured the transcript levels of nine structural genes involved in anthocyanin biosynthesis in infiltrated tobacco leaves by RT-qPCR (Figure 7). We focused on the upstream genes *PHENYLALANINE AMMONIA LYASE* (*NtPAL*) and *4-COUMARATE:COA LIGASE* (*Nt4CL*); the early biosynthetic genes *NtCHS*, *NtCHI*, *NtF3H*, and *NtF3′H*; and the late biosynthetic genes *NtDFR*, *NtANS*, and *NtUFGT*. Transient infiltration of *BrPAP1* alone induced transcription of the anthocyanin biosynthetic genes, including *NtF3′H*, *NtDFR*, and *NtUFGT*, compared to that of the empty vector and *BrTT8*, resulting in a slight accumulation of pigments. *BrTT8* expression did not activate the transcription of anthocyanin biosynthetic genes or result in anthocyanin accumulation, as transcript levels were comparable to those of the control leaves transfected with empty vector. Transient expression of *BrPAP1* with *BrTT8* strongly induced transcription of not only the upstream genes *NtPAL* and *Nt4CL*, but all anthocyanin biosynthetic genes. Co-infiltration of *BrMYBL2.1-G* with *BrPAP1* and *BrTT8* greatly reduced the transcript level of all genes activated by *BrPAP1* and *BrTT8*. Co-infiltration of *BrMYBL2.1-P* with *BrPAP1* and *BrTT8* slightly reduced the transcript levels of anthocyanin biosynthetic genes compared with co-expression of *BrPAP1* and *BrTT8*. However, the repression by *BrMYBL2.1-P* was much lower than that produced by *BrMYBL2.1-G*, in agreement with the anthocyanin contents (Figure 6B). These results indicated that BrMYBL2.1-G, but not BrMYBL2.1-P, participates in anthocyanin biosynthesis as a transcriptional repressor.

### 2.6. BrMYBL2.1-G Actively Inhibits Transcription at the BrCHS and BrDFR Promoters 

**Figure 8:**
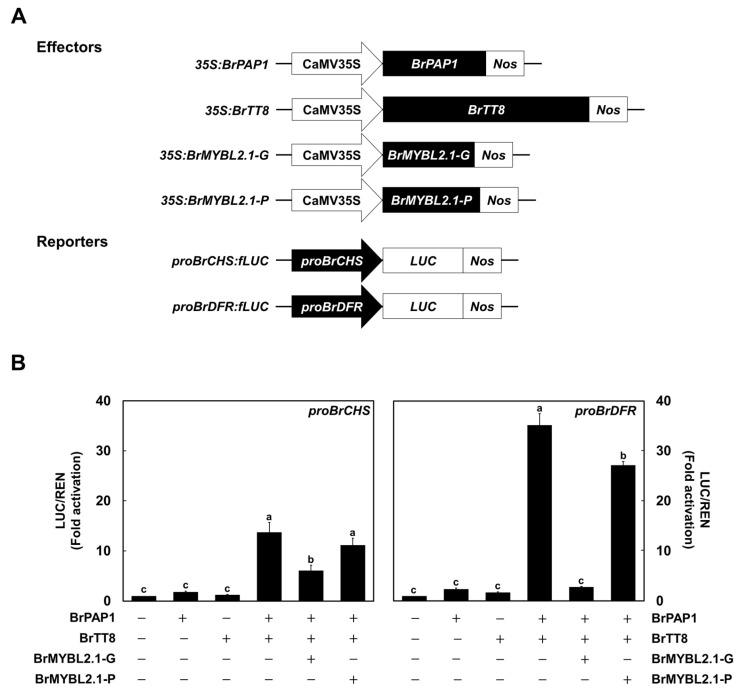
Characterization of the regulatory roles of BrMYBL2.1-G and BrMYBL2.1-P for *BrCHS* and *BrDFR* promoter activation. (**A**) Schematic diagrams of the effector and reporter constructs used in this assay. The effector constructs consist of the coding sequences of *BrPAP1*, *BrTT8*, *BrMYBL2.1-G*, and *BrMYBL2.1-P* driven by the 35S promoter. The reporter constructs contain the firefly luciferase reporter gene (*LUC*) under the control of the *BrCHS* and *BrDFR* promoters. (**B**) Promoter transactivation assays in Arabidopsis protoplasts transiently transfected with the constructs *BrPAP1*, *BrTT8*, *BrMYBL2.1-G*, and *BrMYBL2.1-P* in the indicated combinations. Results are the means ± SD from three independent biological replicates. Different letters indicate significantly different values (*p* < 0.01), as determined by one-way ANOVA followed by Duncan’s multiple range tests.

To directly validate the repressive role of BrMYBL2.1-G in anthocyanin biosynthesis, we conducted a promoter activation assay with the *BrCHS* and *BrDFR* promoters in transiently transfected Arabidopsis protoplasts (Figure 8A). The firefly luciferase reporter gene (*LUC*) was placed under the control of the *BrCHS* or *BrDFR* promoter, and Renilla luciferase gene (*REN*) driven by the *UBIQUITIN10* promoter was used as the internal normalization control. Individual expression with *BrPAP1* or *BrTT8* did not activate the *BrCHS* and *BrDFR* promoters (Figure 8B). However, the simultaneous expression of *BrPAP1* and *BrTT8* dramatically activated the *BrCHS* and *BrDFR* promoters. To investigate the role of *BrMYBL2.1*, the active regulators of *BrPAP1* and *BrTT8* were used together with the *BrMYBL2.1-G* or *BrMYBL2.1-P* construct, and the activation was measured. *BrMYBL2.1-G* was able to dramatically inhibit the transactivation of *BrCHS* and *BrDFR* promoter activated by *BrPAP1* and *BrTT8*. In turn, *BrMYBL2.1-P* slightly repressed the transactivation of the *BrDFR* promoter, but not the *BrCHS* promoter. These results showed that anthocyanin biosynthesis is efficiently repressed by BrMYBL2.1-G, but not by BrMYBL2.1-P. 

### 2.7. BrMYBL2.1 Protein Is Regulated by the Ubiquitin Proteasome System

Previous studies revealed that negative regulators such as FhMYB27 and PtrMYB182 required interaction with bHLH TF of anthocyanin activating MBW complexes [29,37]. Additionally, mutation of C2 and C5 motifs resulted in the loss of repressive activities. These results demonstrated that the underlying mechanism of the negative regulator involves an interaction with the bHLH cofactors and repressive activity. As seen in Figure 5, BrMYBL2.1-P failed to interact with the bHLH cofactors BrTT8 despite harboring intact BIM and repressive motifs. Taken together, these results suggest that the repression activity of BrMYBL2.1 would be severely affected by the variation between BIM and C2 motif. 

**Figure 9:**
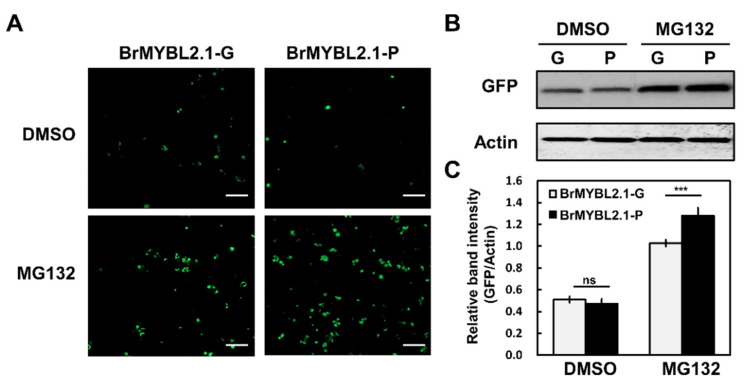
Degradation of BrMYBL2.1-G and BrMYBL2.1-P by the ubiquitin-proteasome system. (**A**) Effect of MG132 on the intensity and localization of GFP fluorescence in transiently transfected Arabidopsis protoplasts. Bars, 100 μm. (**B**) Immunoblot analysis of total proteins from transfected protoplasts treated with dimethyl sulfoxide (DMSO) or MG132. (**C**) Quantification of the GFP bands shown in B, normalized to actin abundance. Significant differences were determined by a Student’s paired *t*-test relative to BrMYBL2.1-G; *** *p* < 0.001.

Indeed, BrMYBL2.1-P cannot repress anthocyanin biosynthesis, unlike BrMYBL2.1-G activity (Figure 6 and Figure 7). BrMYBL2.1-P does carry a stretch of 13 lysine residues, which may be modified via ubiquitination and targeted by the ubiquitin proteasome system. To test this hypothesis, we monitored BrMYL2.1 protein accumulation in Arabidopsis protoplasts transiently transfected with *35S: BrMYBL2.1-G-GFP* or *35S:BrMYBL2.1-P-GFP*, and incubated with dimethyl sulfoxide (DMSO, mock treatment), or the proteasome inhibitor MG132 (dissolved in DMSO). We detected strong GFP fluorescence in protoplasts transfected with either construct in the presence of MG132 (Figure 9A). In the absence of MG132 treatment, green-fluorescent signals from the BrMYL2.1-GFP fusions rapidly declined compared with those of protoplasts treated with MG132. This result suggested that BrMYL2.1 abundance may be controlled by ubiquitination. Immunoblot analysis using an anti-GFP antibody demonstrated that BrMYL2.1-P accumulates to higher levels when the proteasome pathway is blocked, compared with BrMYL2.1-G (Figure 9B,C). These results suggested that the multiple lysine residues in BrMYL2.1-P may be targets for ubiquitin-proteasome-mediated degradation, leading to the degradation of BrMYL2.1-P and preventing it from repressing anthocyanin biosynthesis. 

## 3. Discussion

### 3.1. BrMYBL2.1 Regulates Anthocyanin Biosynthesis

We report here that the R3 MYB TF BrMYBL2.1 contributes to the regulation of anthocyanin biosynthesis in seedlings of Chinese cabbage. Deciphering the regulatory mechanisms behind anthocyanin biosynthesis and its spatiotemporal control has implications for the development of novel plant and fruit varieties with higher anthocyanin contents through conventional or advanced breeding methods.

The ternary MBW complex, comprising R2R3 MYB, bHLH, and WD40-repeat protein, controls the site and timing of anthocyanin biosynthesis [14,35,38]. Small R3 MYB proteins, such as Arabidopsis AtMYBL2, chrysanthemum CmMYB#7, petunia PhMYBx, grape hyacinth (*Muscari armeniacum*) MaMYBx, and tomato (*Solanum lycopersicum*) SIMYB-ATV, negatively regulate the accumulation of anthocyanins, which are responsible for the pigmentation in various organs, including flowers, fruits, and leaves [16,26,27,39,40,41]. These repressors of anthocyanin biosynthesis can be divided into two groups, depending on the presence of repressor motifs [42]. Usually, active repressors contain a BIM in the R3 domain and additional repressive motifs at their C-termini. For instance, freesia FhMYB27, petunia PhMYB27, alfalfa (*Medicago truncatula*) MtMYB2, and Arabidopsis AtMYBL2 are active repressors with C-terminal repressor motifs. Mutations or deletions of these repressor motifs result in the loss of transcriptional repression [16,26,37,42]. Passive repressors only contain the BIM at their N termini. Examples include Arabidopsis AtCPC and tomato SlMYB-ATV, whose loss of function cause ectopic anthocyanin accumulation in plant [18,39]. These proteins appear to act as inhibitors of the MBW complex by competing with MYB activators for binding to the partner bHLH, resulting in the formation of inactive MBW complexes. The interaction between activators and repressors is important in regulating this metabolic pathway.

Anthocyanin biosynthesis was influenced by diverse environmental and biological factors, such as light, temperature, sugar, and hormones [21,43]. As in the previous studies, the transcript level of the active regulators, *BrPAP1* and *BrTT8*, was highly detected under low temperature and sucrose treatment, but that of the negative regulator, *BrMYBL2.1*, was barely expressed in the P cultivar (Figure 2). In contrast to the expression pattern of active regulators, the transcript level of *BrMYBL2.1* in the G cultivar was relatively highly detected in the treatment under optimum temperature and without sucrose. These results indicate that a sophisticated regulating mechanism on activators and repressors contributes to anthocyanin biosynthesis under temperature and sucrose treatment. Sequence alignment and phylogenetic analysis showed that *BrMYBL2.1* is an active repressor for the negative regulation of anthocyanin biosynthesis in Chinese cabbage. The expression pattern of *BrMYBL2.1* was opposite to that of *BrTT8* and most anthocyanin biosynthetic genes, indicating that it may directly repress anthocyanin biosynthesis via its repressor motif. Unlike BrMYBL2.1-G, the BrMYBL2.1-P variant failed to interact with BrTT8 to repress anthocyanin biosynthesis although it has an intact BIM reported to be indispensable for interacting with the bHLH partner; we attribute this to the insertion of 20 amino acids specific to the BrMYBL2.1 allele in the purple cultivar (Figure 5 and Figure S1). These results suggest that the region near the R3 domain is important in shaping the interaction with the bHLH TF. 

Simultaneous co-expression of *BrMYBL2.1-G*, *BrPAP1*, and *BrTT8* prevented the accumulation of anthocyanins and lowered the transcript levels of anthocyanin biosynthetic genes in tobacco leaves. By contrast, *BrMYBL2.1-P* did not inhibit anthocyanin accumulation and activation of the *BrCHS* and *BrDFR* promoters in the same conditions (Figure 6 and Figure 8). 

### 3.2. BrMYBL2.1 Variants Illustrate the Role of a Dysfunctional Protein by Insertional Mutations 

Expression of anthocyanin biosynthetic genes is regulated by rapid turnover of the MBW complex [11]. The TFs assembled into active MBW complexes are post-translationally regulated. For example, Arabidopsis AtPAP1 and AtPAP2, and apple MdMYB1, are ubiquitinated and degraded via the 26S proteasome pathway by the nucleus-localized E3 ubiquitin ligase CONSTITUTIVE PHOTOMORPHOGENIC1 (COP1) in the dark [44,45]. In the light, the COP1 abundance in the nucleus decreases dramatically, allowing the accumulation of active R2R3 MYBs, including AtPAP1, AtPAP2, and MdMYB1. We established that the abundance of BrMYBL2.1-G and BrMYBL2.1-P is post-translationally modulated via ubiquitination and degradation by the 26S proteasome pathway. BrMYBL2.1-P harbored multiple lysine residues that are probable targets for ubiquitination. The degradation of BrMYBL2.1-P would thus contribute to the lack of repression of anthocyanin biosynthesis and explain the accumulation of the pigments in Chinese cabbage cultivars with purple leaves.

**Figure 10:**
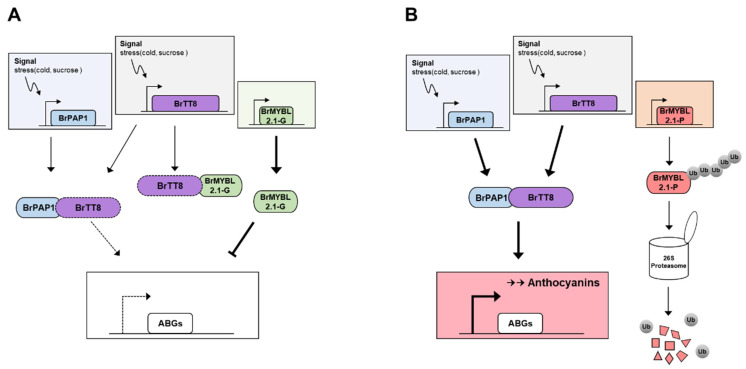
Proposed working model of the role of BrMYBL2.1 in anthocyanin biosynthesis in green (**A**) and purple (**B**) Chinese cabbage. Arrows and blunt arrows represent positive and negative regulation, respectively.

Based on these results, we proposed a model of anthocyanin biosynthesis by repression of BrMYBL2.1 in Chinese cabbage leaves (Figure 10). In the G cultivar, a high level of gene expression and protein stability of BrMYBL2.1 led to repress the expression of anthocyanin biosynthetic genes and interference in the formation of an active MBW complex via interacting with BrTT8, resulting in low anthocyanin accumulation. In turn, the high level of anthocyanins detected in the P cultivar can be explained by the dysfunctional capacity of BrMYBL2.1-P, suggesting that not only disability to interaction with BrTT8 but also protein stability affected the induction of anthocyanin biosynthesis. The defect in repressor protein can enhance the interacting ability of BrTT8 into BrPAP1, causing the higher expression level of anthocyanin biosynthetic genes and the anthocyanin accumulation.

The role of BrMYBL2.1 in anthocyanin biosynthesis also provides a potential target for genetic engineering via genome editing approaches such as clustered regularly interspaced short palindromic repeats/Cas9 system to enhance anthocyanin contents in edible plant tissues, as well as to reduce anthocyanin degradation. 

## 4. Materials and Methods

### 4.1. Plant Materials

Two cultivars of Chinese cabbage seedlings, green-leaved (G) ‘Bulam 3’ (Farm Hannong Seed Co., Seoul, Korea) and purple-leaved (P) ‘8267’ (Asia Seed Co., Seoul, Korea), were used in this study. Seeds from each cultivar were surface-sterilized using 70% (*v*/*v*) ethanol and 0.01% (*v*/*v*) Triton X-100 before being sown onto half-strength Murashige and Skoog (MS) medium (Duchefa, Haarlem, The Netherlands), with or without 90 mM sucrose. After stratification for 2 days at 4 °C, plates were released into long-day light conditions (16-h-light/8-h-dark) and incubated at 24 °C for 4 days, or incubated at 24 °C for 3 days followed by incubation at 4 °C for 24 h. Tobacco (*Nicotiana tabacum* cv. Xanthi) plants grown in greenhouses at Hankong University (Anseong, Korea) under natural light at 26  ±  2 °C were used for transient Agrobacterium (*Agrobacterium tumefaciens*)-mediated infiltration assays. 

Anthocyanin contents and the transcript levels of anthocyanin-related genes were analyzed from the seedlings of the two Chinese cabbage cultivars grown under the above-mentioned cultivation conditions and from transiently infiltrated tobacco leaves. All samples were rapidly frozen in liquid nitrogen and stored at −80 °C. Each sample was then ground to powder and split into two aliquots, one for RNA extraction and the other to determine the anthocyanin contents.

### 4.2. RNA Extraction, cDNA Synthesis, and Isolation of Genomic DNA 

Total RNA was extracted from the seedling of Chinese cabbage and from the leaves of tobacco using TRIzol reagent (Invitrogen, Carlsbad, CA, USA) and purified using the FavorPrep Plant Total RNA Mini Kit (Favorgen, Changzhi, Taiwan), according to the manufacturer’s instructions. First-strand cDNA was synthesized from 2 μg total RNA using the amfiRivert cDNA Synthesis Platinum Master Mix (GenDEPOT, Barker, TX, USA).

Genomic DNA was extracted from the leaves of Chinese cabbage using the Plant Mini Kit (Qiagen, Valencia, CA, USA) according to the manufacturer’s instructions.

### 4.3. Measurement of Total Anthocyanin Contents 

Total anthocyanin contents from the seedlings of Chinese cabbage and from the transiently infiltrated leaves of tobacco were determined according to a previously described method [13]. Samples were ground to powder in liquid nitrogen. Aliquots of 100 mg fresh weight were then mixed in 600 μL extraction buffer (methanol containing 1% [*v*/*v*] HCl) for 6 h at 4 °C with moderate agitation. Addition of 200 μL water and 200 μL chloroform was followed by centrifugation to pellet plant debris at 14,000× *g* for 5 min at 4 °C. After centrifugation, absorbance of the supernatant was recorded at 530 nm (A_530_) and 657 nm (A_657_) using a microplate reader. Anthocyanin contents were determined according to the formula A_530_ − (0.25 × A_657_). Each sample was extracted from three independent experiments and measured.

### 4.4. Reverse Transcription-Quantitative PCR (RT-qPCR Analysis)

Transcript levels were determined by RT-qPCR using the AccuPower 2 × Greenstar qPCR Master Mix (Bioneer, Daejun, Korea) and a Bio-Rad CFX96 Detection System (Bio-Rad Laboratories, Hercules, CA, USA) according to the manufacturer’s instructions. Gene expression levels were normalized to the *ELONGATION FACTOR 1 ALPHA (BrEF1α)* and *GLYCERALDEHYDE 3-PHOSPHATE DEHYDROGENASE (NtGAPDH)* for Chinese cabbage and tobacco, respectively, as the reference gene. Three independent biological replicates were performed. The primers used for RT-qPCR analysis are listed in Supplementary Table S1.

### 4.5. Gene Cloning and Sequence Analysis

The full-length coding sequences of *BrMYBL2.1* were amplified from cDNA and genomic DNA derived from G and P Chinese cabbage plants by PCR with PrimeSTAR HS DNA Polymerase (Takara, Otsu, Japan) using the primer pair BrMYBL2.1-F/R (Supplementary Table S1). All amplicons were subcloned into the pENTR/D-TOPO vector (Invitrogen) for validation by sequencing.

The nucleotide sequences, deduced protein sequences, and open reading frames (ORFs) of *BrMYBL2.1* from the G and P plants were analyzed online (http://www.ncbi.nlm.nih.gov, accessed on 1 February 2022). Structural analysis of the predicted proteins was carried out at the ExPASy Molecular Biology Server (http://cn.expasy.org/tools/, accessed on 1 February 2022). Multiple sequence alignments were generated using the CLUSTALW program (https://www.genome.jp/tools-bin/clustalw, accessed on 1 February 2022). A phylogenetic tree was constructed using the neighbor-joining method [46] with MEGA version 6 software [47].

### 4.6. Subcellular Localization Assay

The subcellular localization of BrMYBL2.1-G and BrMYBL2.1-P was analyzed in Arabidopsis protoplasts as previously described [13]. GFP fusion constructs were generated in the p326-sGFP plasmid, which contains the cauliflower mosaic virus 35S promoter. For C-terminal GFP fusions, the coding sequences of *BrMYBL2.1-G* and *BrMYBL2.1-P* were individually amplified using gene-specific primer sets (p326-BrMYBL2.1-F/R), which introduced an *Xba*I restriction site upstream of the ATG codon, and cloned in-frame and downstream of *GFP* using the In-Fusion HD Cloning Kit (Takara). The resulting p326-BrMYBL2.1-G and p326-BrMYBL2.1-P constructs were sequenced to confirm the absence of errors during PCR amplification. The plasmids were transfected into Arabidopsis mesophyll protoplasts via polyethylene glycol (PEG)-mediated transformation. After incubation for 16–20 h at 25 °C in the dark, images were captured by fluorescence confocal microscopy (Leica TCS SP8, Leica Microsystems, Wetzlar, Germany).

### 4.7. Yeast Two-Hybrid (Y2H) Assays

Full-length coding sequences of *BrMYBL2.1-G*, *BrMYBL2.1-P*, *BrPAP1* and *BrTT8* were individually amplified using specific primer sets to generate yeast two-hybrid constructs (Supplementary Table S1). The amplified fragments were then cloned in-frame with the sequences encoding the AD into pGADT7 and the BD into pGBKT7, respectively, using the In-Fusion Cloning System (Takara). 

The AD and BD constructs were co-transformed into yeast (*Saccharomyces cerevisiae*) strain MaV203 following the manufacturer’s instructions (Takara). Yeast colonies were selected on SD medium lacking Trp and Leu and were replicated onto SD medium lacking Trp, Leu, and His and containing 10 mM 3-amino-1,2,4-triazole, a competitive inhibitor of the *HIS3* gene product. The plates were photographed after 2 days of incubation in the dark at 30 °C.

### 4.8. In Planta Assays of BrMYBL2.1 Function in Anthocyanin Biosynthesis

The plasmid used for transient infiltration assays of tobacco leaves was constructed as follows. The cDNA sequences of *BrMYBL2.1-G*, *BrMYBL2.1-P*, *BrPAP1* and *BrTT8* were amplified with specific primer sets (Supplementary Table S1) and cloned into the pENTR/D-TOPO vector (Invitrogen) before incorporation into the Gateway destination vector pB2GW7 (VIB-Ghent University, Ghent, Belgium) through several Gateway cloning steps. The resulting constructs were introduced into *Agrobacterium* strain GV3101 for transient infiltration of the abaxial leaf surface of tobacco plants. Leaf color was monitored 5 days later, as described [32].

### 4.9. Promoter Activation Assays

For transcriptional activity assays, the coding sequences of *BrMYBL2.1-G*, *BrMYBL2.1-P*, *BrPAP1*, and *BrTT8* were inserted in place of *sGFP* into the p326-sGFP plasmid digested with *Xba*I and *Not*I to generate effector constructs. For the firefly luciferase (*LUC*) reporter constructs, *BrCHS* and *BrDFR* promoter regions were PCR-amplified from genomic DNA isolated from the purple Chinese cabbage leaves and were inserted into the p326-LUC vector. The reporter constructs also contained the *Renilla* luciferase (*REN*) gene driven by the *UBIQUITIN10* promoter as an internal control.

Isolation and transient transfection of Arabidopsis protoplasts were performed as previously described [29]. The activity of LUC and REN was measured using a dual-luciferase assay system (Promega, Madison, WI, USA), according to the manufacturer’s protocol. Normalized reporter activity was calculated as the LUC/REN ratio; this ratio was then set to 1 for transient transfection in the absence of effector.

### 4.10. Protein Accumulation Assay for BrMYBL2.1s in Arabidopsis Protoplasts

Protoplasts were prepared from Arabidopsis seedlings. The vectors *35S:BrMYBL2.1-G* and *35S:BrMYBL2.1-P* were introduced into Arabidopsis protoplasts via PEG-mediated transformation, as described previously [28]. After 4 h, 100 μM MG132 was added to the protoplasts and they were incubated for 24 h. GFP fluorescence was examined under a confocal laser scanning microscope (Olympus FV300, Olympus, Berlin, Germany).

Protein extracts were prepared in ice-cold extraction buffer (150 mM NaCl, 50 mM Tris-HCl, pH 7.5, 1 mM EDTA, 0.5% [*v*/*v*] NP-40, 1 mM PMSF, 50 μM MG132, and one protease inhibitor cocktail tablet per 10 mL (Roche, Mannheim, Germany)). Total proteins were separated using 12% SDS-PAGE gels. Immunoblots were performed with an anti-GFP antibody (A02021, Abbkine, Wuhan, China), with an anti-actin antibody (CSB-PA000352, Cusabio Technology LLC, Houston, TX, USA) as a loading control.

## 5. Conclusions

We have demonstrated that anthocyanin biosynthesis is determined, in part, by the negative regulator *BrMYL2.1*. Sequence analysis of *BrMYBL2.1-P* in purple Chinese cabbage revealed an insertion in the third exon of the gene, leading to the introduction of multiple lysine residues near an intact R3 domain. In purple Chinese cabbage, anthocyanin contents and anthocyanin biosynthetic gene expression followed an opposite pattern in a *BrMYL2.1*-dependent manner. A transient expression assay in tobacco leaves demonstrated that *BrMYBL2.1-G* is an active repressor of anthocyanin biosynthesis, whereas *BrMYBL2.1-P* appears to be non-functional. Likewise, promoter activation assays showed that *BrMYBL2.1-G*, but not *BrMYBL2.1-P*, actively represses transcription from the *BrCHS* and *BrDFR* promoters when co-expressed with *BrPAP1* and *BrTT8*. Taken together, these results suggest that the *BrMYBL2.1-G* allele is key in shaping anthocyanin accumulation in the leaves of Chinese cabbage.

## Data Availability

Data supporting the findings of this study are available from the corresponding author, Sun-Hyung Lim, upon request.

## Supplementary Materials

The following supporting information can be downloaded at: https://www.mdpi.com/article/10.3390/ijms23063382/s1.
