# Supplementary material for: The R3-Type MYB Transcription Factor BrMYBL2.1 Negatively Regulates Anthocyanin Biosynthesis in Chinese Cabbage (Brassica rapa L.) by Repressing MYB–bHLH–WD40 Complex Activity
Source: Int J Mol Sci. 2022 Mar 21;23(6):3382. doi: 10.3390/ijms23063382 (PMC8949199; doi:10.3390/ijms23063382)
Supplement: Supplementary file 1 [file ijms-23-03382-s001.zip › For-IJMS_BrMYBL2-1_supp.pdf]

Supplementary Table S1. List of specific primers used in this study.

Usage	Primer Name	Primer Sequence
Gene expression	qRT-BrMYBL2.1-F	ATGGGAATCGATCCAACTAA
	qRT-BrMYBL2.1-R	TAGCATTAGCCTCAGAGCAA
	qRT-BrPAP1-F	GAGAAATATGAGCTCGTTAA
	qRT-BrPAP1-R	TAGCTTTTCTGTCTCTGTAGA
	qRT-BrTT8-F	GCCAAAATTCAGACAGTGGT
	qRT-BrTT8-R	TCTTCTTCGTGCTCTTCGTT
	qRT-BrCHS-F	TCCTTCCTATATGCCTACCTAC
	qRT-BrCHS-R	AAGGAGAGAAAGACAGAATTA
	qRT-BrCHI-F	ATCCGTCCCTTTCTTCCG
	qRT-BrCHI-R	ACTGCTTTAGCCTCAGAGTATGTG
	qRT-BrF3H-F	GAACCATTTTGATGAGAAAGCA
	qRT-BrF3H-R	GAACCTCCCGTTGCTCAGATAG
	qRT-BrF3'H-F	TGCCCGCACTTGATTGTTT
	qRT-BrF3'H-R	CCGTTCTTCATCGCCTCGT
	qRT-BrDFR-F	GACGGCGTTTTCCACATAG
	qRT-BrDFR-R	TCCCCAACACTCCATTCAC
	qRT-BrANS-F	GCAAAATGTGTTCCTGATTCG
	qRT-BrANS-R	ATCTTATCCTTTGGTGGTTC
	qRT-BrUFGT-F	CCCTAACTCTTGTCCCTGCTG
	qRT-BrUFGT-R	AAGTTCTGTTCAAACTTGCTG
	qRT-BrEF1α-F	ATACCAGGCTTGAGCATACCG
	qRT-BrEF1α-R	GCCAAAGAGGCCATCAGACAA
	qRT-NtPAL-F	ATTGAGGTCATCCGTTCTGC
	qRT-NtPAL-R	ACCGTGTAACGCCTTGTTTC
	qRT-Nt4CL-F	TCATTGACGAGGATGACGAG
	qRT-Nt4CL-R	TGGGATGGTTGAGAAGAAGG
	qRT-NtCHS-F	TTGTTCGAGCTTGTCTCTGC
	qRT-NtCHS-R	AGCCCAGGAACATCTTTGAG
	qRT-NtCHI-F	GTCAGGCCATTGAAAAGCTC

	qRT-NtCHI-R	CTAATCGTCAATGCCCCAAC
	qRT-NtF3H-F	CAAGGCATGTGTGGATATGG
	qRT-NtF3H-R	TGTGTCGTTTCAGTCCAAGG
	qRT-NtF3'H-F	AGGCTCAACACTTCTCGT
	qRT-NtF3'H-R	CATCAACTTTGGGCTTCT
	qRT-NtDFR-F	AACCAACAGTCAGGGGAATG
	qRT-NtDFR-R	TTGGACATCGACAGTTCCAG
	qRT-NtANS-F	TGGCGTTGAAGCTCATACTG
	qRT-NtANS-R	GGAATTAGGCACACACTTTGC
	qRT-NtUFGT-F	CAATGTTTGGGATGGTGTCA
	qRT-NtUFGT-R	TTCCTCCTCTGCCTCTTTCA
	qRT-NtGAPDH-F	GGTGTCCACAGACTTCGTGG
	qRT-NtGAPDH-R	GACTCCTCACAGCAGCACCA
Gene cloning	BrMYBL2.1-F	ATGAACAAAATTAGCCACGGCGCTCT
	BrMYBL2.1-R	TCACTGAAAAAGAAGAAGTGTTTCTTGACTC
	BrPAP1-F	ATGGAGGGTTCGCCAAAAGGGTTGAGAAAAG
	BrPAP1-R	GAAACACTAATCAAGTTCTACAGTCTCTCCA
	BrTT8-F	GGGAAAGATGGATGAATTAAGTATTATACCGT
	BrTT8-R	AGAATCTCGGAACTAGAGTTTATTATTATATATG
Subcellular localization	p326-BrMYBL2.1-F	CACGGGGGACTCTAGAATGAACAAAATTAGCCA
	p326-BrMYBL2.1-R	CCATGGATCCTCTAGACTGAAAAAGAAGAAGTG
	p326-BrPAP1-F	CACGGGGGACTCTAGAATGGAGGGTTCGCCAA
	p326-BrPAP1-R	CCATGGATCCTCTAGAATCAAGTTCCACGGTCT
	p326-BrTT8-F	CACGGGGGACTCTAGAATGGATGAATTAAGTAT
	p326-BrTT8-R	CCATGGATCCTCTAGAGAGTTTATTATTATATATG
Yeast two hybrid	pGADT7-BrMYBL2.1-F	GGAGGCCAGTGAATTCATGAACAAAATTAGCCA
	pGADT7-BrMYBL2.1-R	CACCCGGGTGGAATTCTCACTGAAAAAGAAGAA
	pGADT7-BrPAP1-F	GGAGGCCAGTGAATTCATGGAGGGTTCGCCAA
	pGADT7-BrPAP1-R	CACCCGGGTGGAATTCATCAAGTTCCACGGTCT
	pGADT7-BrTT8-F	GGAGGCCAGTGAATTCATGGATGAATTAAGTAT
	pGADT7-BrTT8-R	CACCCGGGTGGAATTCGAGTTTATTATTATATATG
	pGBKT7-BrMYBL2.1-F	CATGGAGGCCGAATTCATGAACAAAATTAGCCA

	pGBKT7-BrMYBL2.1-R	GGATCCCCGGGAATTCTCACTGAAAAAGAAGAA
	pGBKT7-BrPAP1-F	CATGGAGGCCGAATTCATGGAGGGTTCGCCAA
	pGBKT7-BrPAP1-R	GGATCCCCGGGAATTCATCAAGTTCCACGGTCT
	pGBKT7-BrTT8-F	CATGGAGGCCGAATTCATGGATGAATTAAGTAT
	pGBKT7-BrTT8-R	GGATCCCCGGGAATTCCTAGAGTTTATTATTAT
Transient assay	pENTR-BrMYBL2.1-F	CACCATGAACAAAATTAGCCACGGCGCTCT
	pENTR-BrMYBL2.1-R	TCACTGAAAAAGAAGAAGTGTTTCTTGAC
	pENTR-BrPAP1-F	CACCATGGAGGGTTCGCCAAAAGGGTTGAG
	pENTR-BrPAP1-R	CTAATCAAGTTCCACGGTCTCACCATCT
	pENTR-BrTT8-F	CACCCTAATCAAGTTCCACGGTCTCACCATCT
	pENTR-BrTT8-R	CTAGAGTTTATTATTATATATGATTTGAT
Promoter activation	pBrCHS-F	AAGAGTTATCCTCTGTATTCTCCGGTA
	pBrCHS-R	ATCACCATGTTTTACAAGAGTTTGATAGAT
	pBrDFR-F	TAAGAAGAGGAGTAAGCGAGTGAGGAGATA
	pBrDFR-R	AGCTACCATCTTTGTGTGTGAAAGATGGA
	pBrCHS-fLUC-F	AGGCTCTAGAGGATCCAAGAGTTATCCTCTGTATTC
	pBrCHS-fLUC-R	TTGGCGTCTTCCATGGGTTTTACAAGAGTTTGATAG
	pBrDFR-fLUC-F	AGGCTCTAGAGGATCCTAAGAAGAGGAGTAAGCGAGT
	pBrDFR-fLUC-R	TTGGCGTCTTCCATGGCTTTGTGTGTGAAAGATGGATTA

```
                                   1st exon
BrMYB2.1     : ATGAACAAAATTAGCCACGGCGCTCTCTCTCGGCCTTCCG-------------------------------------------------------------------------------- :  40
BrMYBL2.1_G  : ATGAACAAAATTAGCCACGGCGCTCTCTCTCGGCCTTCCGGTAACGTTTCTTGTTCCATATTGTGTATTGTCTTTCATATGACCAAATTCTTCATAATTGAAGATCGGTATAGAAGTCAT : 120
BrMYBL2.1_P  : ATGAACAAAATTAGCCACGGCGCTCTCTCTCGGCCTTCCGGTAACGTTTCTTGTTCAATATTGTGTATTGTCTTTCATATGACCAAATTCTTCATAATTGAAGATCGGTATAGAAGTCAT : 120

                                                                                                                2nd exon
BrMYB2.1     : --------------------------------------------------------------------------------------GAATGCTGCACCGTGCCAAGAGGTATAGAGGGAG :  74
BrMYBL2.1_G  : AGATTACATATATGTACATGTGCACGAGTGAGTTTGCAACAAATGTCGTTTTACTTTGTGAAATTTAATCCCTAATCATGTTTTAGGAATGCTGCACCGTGCCAAGAGGTATAGAGGGAG : 240
BrMYBL2.1_P  : AGATTACATATATGTACATGTGCACGAGTGAGTTTGCAACAAATGTCGTTTTACTTTGTGAAATTTAATCCCTAATCATGTTTTAGGAATGCTGCACCGTGCCAAGAGGTATAGAGGGAG : 240

BrMYB2.1     : AAAGTACGCAAAGCCAGAACTTAAAGAAAGCAACTTCTCAAAAGACGAGGACGATCTCATCCTCAAGCTTCATGCACTTCTTGGCAATAG------------------------------ : 164
BrMYBL2.1_G  : AAAGTACGCAAAGCCAGAACTTAAAGAAAGCAACTTCTCAAAAGACGAGGACGATCTCATCCTCAAGCTTCATGCACTTCTTGGCAATAGGTTTTCATTCTTCTTCTTTAATTCAATTTC : 360
BrMYBL2.1_P  : AAAGTACGCAAAGCCAGAACTTAAAGAAAGCAACTTCTCAAAAGACGAGGACGATCTCATCCTCAAGCTTCATGCACTTCTTGGCAATAGGTTTTCATTCTTCTTCTTTAATTCAATTTC : 360

                                                                                                       3rd exon
BrMYB2.1     : ----------------------------------------------------------------------ATGGTCACTGATAGCGGGAAGATTGCCTGGACGAACCGACGACGAAGTAA : 214
BrMYBL2.1_G  : GAGCGTTATTATAAAAATATACCATAATGTGAATGTTTGTAATAATAAGATTTTGATGGTGCGCTTTCAGATGGTCACTGATAGCGGGAAGATTGCCTGGACGAACCGACGACGAAGTAA : 480
BrMYBL2.1_P  : GAGCGTTATTATAAAAATATACCATAATGTGAATGTTTGTAATAATAAGATTTTGATGGTGCGCTTTCAGATGGTCACTGATAGCGGGAAGATTGCCTGGACGAACCGACGACGAAGTAA : 480

BrMYB2.1     : GGATCCATTGGGAAAGTTACTTAGAGAAGAAACTCATGAAAAT------------------------------------------------------------GGGAATCGATCCAACTA : 274
BrMYBL2.1_G  : GGATCCATTGGGAAAGTTACTTAGAGAAGAAACTCATGAAAAT------------------------------------------------------------GGGAATCGATCCAACTA : 540
BrMYBL2.1_P  : GGATCCATTGGGAAATTTACTTAGAGAAGAAACTCATGAAAATGGGAATCGATCCAACTTTGTTTCAAAAAAAAAAAAAAAAAAAAAAAAAAAAAAAAAAAAAGGGAATCGATCCAACTA : 600

BrMYB2.1     : ATCATCGTATCTACCATCACACCAACTACACTTCTAGACGATTCATCAATGCCTCGTATAAGAAACATGAAACCGATATTATTAGTGATCAATCTTCTTCGGTATCTGAATCATGTGATA : 394
BrMYBL2.1_G  : ATCATCGTATCTACCATCACACCAACTACACTTCTAGACGATTCATCAATGCCTCGTATAAGAAACATGAAACCGATATTATTAGTGATCAATCTTCTTCGGTATCTGAATCATGTGATA : 660
BrMYBL2.1_P  : ATCATCGTATCTACCATCACACCAACTACACTTCTAGACGATTCGTCAATGCCTCGTATAAGAAACATGAAACCGATATTATTAGTGATCAATCTTCTTCGGTATCTGAATCATGTGATA : 720

BrMYB2.1     : TGAAACTATTACCCGTTTCAAGTACCAATTGCTCTGAGGCTAATGCTAGTTCTGGAAACAGCCGGTTGCCTGACCTCAACATCGGTCTCGTCCCGATAAAGACCGTGACTTCTTTGCCAG : 514
BrMYBL2.1_G  : TGAAACTATTACCCGTTTCAAGTACCAATTGCTCTGAGGCTAATGCTAGTTCTGGAAACAGCCGGTTGCCTGACCTCAACATCGGTCTCGTCCCGATAAAGACCGTGACTTCTTTGCCAG : 780
BrMYBL2.1_P  : TGAAACTATTACCCGTTTCAAGTACCAATAGCTCTGAGGCTAATGCTAGTTCTGGAAACAGCCGGTTGCCTGACCTCAACATCGGTCTCGTCCCGATAAAGACCGTGACTTCTTTGCCAG : 840

BrMYB2.1     : TTGGCTCCCTTCAAGAACCTAGCGGATCCTCTAACCATGGTTCAACGAGTCAAGAAACACTTCTTCTTTTTCAGTGA : 591
BrMYBL2.1_G  : TTGGCTCCCTTCAAGAACCTAGCGGATCCTCTAACCATGGTTCAACGAGTCAAGAAACACTTCTTCTTTTTCAGTGA : 857
BrMYBL2.1_P  : ATGGCTCCCTTCAAGAACCTAGCGGATCCTCTAACCATGGTTCAACGAGTCAAGAAACACTTCTTCTTTTTCAGTGA : 917
```

Supplementary Figure S1. Multiple alignments of the genomic sequence of *BrMYBL2.1* derived from green (*BrMYBL2.1-G*) and purple (*BrMYBL2.1-P*) Chinese cabbage and the previously reported *BrMYBL2.1* sequence. The start and stop codons are indicated by the blue and red boxes, respectively. Exons are indicated by gray boxes. Target site duplication is highlighted in green color. Poly (A) insertion is marked by red letters.

```
                          *        20         *        40         *
BrPAP1_G : MEGSPKGLRKGAWTAEEDSLLRQCIDKYGEGKWHQVPLRAGLNRCRKSCR :  50
BrPAP1_P : MEGSPKGLRKGAWTAEEDSLLRQCIDKYGEGKWHQVPLRAGLNRCRKSCR :  50

                         60         *        80         *       100
BrPAP1_G : LRWLNYLKPSIKKGKLSSDEVDLLLRLHKLLGNRWSLIAGRLPGRTANDV : 100
BrPAP1_P : LRWLNYLKPSIKKGKLSSDEVDLLLRLHKLLGNRWSLIAGRLPGRTANDV : 100

                          *       120         *       140         *
BrPAP1_G : KNYWNTHLSKKHEPGCNTKMRKRNIPCSSTQPAQKNEVLKPRPRSFTVNN : 150
BrPAP1_P : KNYWNTHLSKKHEPGCNTKMRKRNIPCSSTQPAQKNEVLKPRPRSFTVNN : 150

                        160         *       180         *       200
BrPAP1_G : GCSHFNGKPKVDVIPLFLGVNNTNNVCENSITYKKDAEKYELVNNLMDGE : 200
BrPAP1_P : GCSHFNGQPKVDVIPLFLGVNNTNNVCENSITYKKDAEKYELVNNLMDGE : 200

                          *       220         *       240
BrPAP1_G : NMWWKSLLEESQEPDAIVPESTETEKLATSAFDVEQLWNLLDGETVELD : 249
BrPAP1_P : NMWWKSLLEESQEPDAIVPESTETEKLATSAFDVEQLWNLLDGETVELD : 249
```

Supplementary Figure S2. Multiple alignments of the amino acid sequences of BrPAP1 derived from green (BrPAP1-G) and purple (BrPAP1-P) Chinese cabbage. The alignment between BrPAP1-G and BrPAP1-P revealed the one amino acid substation at position 158.
